# The Impact of Ideal Cardiovascular Health Behaviors on Mental Health and Well-Being Among Baltic Countries Adolescents: Findings from the HBSC Study

**DOI:** 10.3390/ijerph22040491

**Published:** 2025-03-25

**Authors:** Rafaela C. Espírito Santo, Geiziane R. Melo, Viney P. Dubey, Cesar Agostinis-Sobrinho

**Affiliations:** Health Research and Innovation Science Centre, Faculty of Health Sciences, Klaipeda University, LT-92294 Klaipeda, Lithuania; geiziane.melo@ku.lt (G.R.M.); vineydreamz@gmail.com (V.P.D.); cesaragostinis@hotmail.com (C.A.-S.)

**Keywords:** epidemiology, lifestyle, exercise, diet, ideal cardiovascular health, psychosomatic disorder

## Abstract

This study explored the association between ideal cardiovascular health behaviors (ICHBs) and mental health and well-being in Baltic adolescents using data from the 2018 HBSC survey. ICHBs included smoking status, body mass index, physical activity, and healthy diet adherence. Mental health included low mood, irritability, nervousness, and sleep difficulties, while well-being included life satisfaction and self-reported health. Multi-regression analyses were performed. The study included 12,934 adolescents (mean age: 13.6 ± 1.6 years). Non-smokers, non-overweight individuals, and physically active participants were more likely to report good mental health, higher life satisfaction, and better self-reported health. Adherence to a healthy diet was linked to improved self-reported health and greater life satisfaction. Compared to those with four ICHBs (reference), adolescents with only one ICHB had higher odds of feeling low (OR = 0.28, 95% CI: 0.13–0.62), irritability (OR = 0.35, 95% CI: 0.19–0.66), nervousness (OR = 0.26, 95% CI: 0.13–0.55), and sleep difficulties (OR = 0.29, 95% CI: 0.14–0.61). Adolescents with two ICHBs had higher odds of feeling low (OR = 0.45, 95% CI: 0.20–0.97) and nervousness (OR = 0.40, 95% CI: 0.19–0.83). These findings highlight the importance of promoting multiple ideal health behaviors to improve mental health and well-being among adolescents.

## 1. Introduction

Cardiovascular diseases are among the leading causes of death in adults worldwide [1]. It is recognized that adolescence is a crucial period of development during which lifestyle habits are acquired and may persist into adulthood [2]. Thus, acquiring healthy lifestyle habits during adolescence can reduce mortality into adulthood [3].

Building upon this understanding, the American Heart Association (AHA) has published metrics to monitor cardiovascular health [4] in which ideal cardiovascular health behavior (ICHB) for children and adolescents was considered by the presence of both ideal health behaviors (non-smoking behavior, body mass index < 85th Percentile, and ≥60 min of moderate- or vigorous-intensity activity every day) and adherence to a healthy diet. The ICHB is associated with gender, age [5,6], education [6], cardiorespiratory fitness [7], and quality of life in adolescents [8].

In addition to cardiovascular health, mental disorders have become a concern for health policymakers. In 2018, Health at a Glance reported that one in six people in Europe (EU) suffered from mental health issues [9]. Interestingly, the Baltic countries (Estonia, Latvia, and Lithuania) had the highest mental health problems (above 17.3%) prevalence in relation to Europe overall. Currently, the State of Health in the EU Report 2023 indicates that the overall Europe of chronic depression is approximately 6% for males and 9% for females [10]. Males from Estonia and Lithuania exhibit a lower rate of chronic depression, whereas males from Latvia show a higher rate of chronic depression compared to the overall European average. On the other hand, females from Estonia and Lithuania exhibited a similar percentage of chronic depression (approximately 9%) compared to the overall European average. However, females from Latvia showed the highest percentage of chronic depression compared to the overall European average, exceeding 10% [10].

Data from the World Health Organization (WHO) on adolescents indicate that as age increases, there is a decline in mental well-being and life satisfaction [11]. In the Baltic countries, adolescents have similar historical and social backgrounds [12], with a high prevalence of mental health problems due to the socio-political context of the post-Soviet transition that led to excessive use of cigarettes and alcohol in young adults and the elderly [13]. Indeed, these findings unequivocally underscore the importance of mental health management, especially in the Baltic countries.

Considering the high risk of mental disorders worldwide [14] and the possibility that ICHBs predict mental disorders, studies have demonstrated that poorer cardiovascular health is associated with poorer mental disorders in adults [15,16]. In adolescents, a previous study involving two UK birth cohort studies demonstrated that smoking, alcohol use, and higher BMI were more likely to co-occur with mentally ill health [17]. Recently, a study from the Canadian HBSC Surveys showed that screen time (ST) levels were positively associated, while physical activity (PA) levels were negatively associated, with reporting frequent psychosomatic complaints in a dose-dependent manner [18]. To our knowledge, no study has examined the association between the accumulation of ICHBs and mental health or well-being in adolescents from the Baltic countries. It is important to assess the association between the accumulation of ICHBs and mental health or well-being in adolescents from the Baltic countries given that (1) the burden of mental health-related mortality remained high and showed divergent temporal changes across the Baltic countries and fractions of various external causes of death and alcohol-related causes of death should be considered in assessing the total burden of mental health-related mortality [13]; (2) adolescents from the Baltic countries share similar historical and social backgrounds [12], with a high prevalence of mental health problems linked to the socio-political context of the post-Soviet transition, which contributed to excessive cigarette and alcohol use from young adulthood to old age in the region [13]; and (3) the suicide rate in the Baltic countries, particularly in Lithuania, is higher than in other European countries [13,19]. 

Additionally, it is important that the WHO Health 2020 policy framework defines early and targeted promotions of well-being and mental health as playing an essential role in continuous health monitoring as a central strategy that contributes to the healthy development of children and future generations [20]. Therefore, the aim of this study was to examine the association between the accumulation of ICHBs and mental health or well-being in adolescents from Baltic Countries. We hypothesize that the complex interplay of biological, psychological, social, and behavioral mechanisms (ICHBs) can contribute to mental health issues, and they often interact in ways that exacerbate the overall impact on an adolescent’s well-being.

## 2. Materials and Methods

This is a cross-sectional study based on Baltic regional data from the 2018 wave of the Health Behaviors in School-aged Children (HBSC) study [21].

Participants and procedures: The HBSC is an international survey used to describe the status of adolescent mental health and well-being across a range of indicators, the role of gender, age, and social inequality, and how adolescent mental health and well-being have changed over time. Data were collected in all participating countries and regions through school-based surveys using the standard methodology detailed in the HBSC international study protocol [21]. The HBSC survey protocol was approved by the Institutional Ethical Board of the National Institute of Health (PRE-876/17). Children’s rights were specifically protected through the United Nations Convention on the Rights of the Child [22]. Documentation was provided to inform parents and children about how confidentiality and anonymity were ensured, detailing who had access to the data and how it was stored and used. Explanations were given in a way that children could understand. Parental (or guardian) and pupil consent was obtained, as the young people involved were generally under the age of legal consent. The approach typically adopted in HBSC was ‘opt-out’ or ‘passive’ consent, allowing participants the option to withdraw. In each country, the study procedures were approved by institutional review boards [21].

## 3. Measures

Cardiovascular health behavior. According to the American Heart Association’s definition, four key cardiovascular health behaviors—body mass index, smoking status, physical activity, and diet—were evaluated [4]. The criteria for ideal cardiovascular health behavior metrics were determined based on the association’s guidelines for children and adolescents aged 5 to 19 years [4]. The Ideal Cardiovascular Health Behavior (ICHB) score was assigned by awarding 1 point for each cardiovascular health behavior at an ideal level, resulting in a total score ranging from 0 to 4 [4].

Smoking behavior. Adolescents were asked to state the number of days they had smoked in the last 30 days, the number of cigarettes per week (“every day”, “at least once a week but not every day”, “less than once a week”, and “I do not smoke”), and the frequency of smoking in the last 30 days (“not at all”, less than one cigarette per week”, “less than one cigarette per day”, 1–5 cigarettes per day”, “6–10 cigarettes per day”, “11–20 cigarettes per day”, and “more than 20 cigarettes per day”). These questions have shown satisfactory validity and reliability in previous studies [23]. We considered non-smokers or the ideal health behavior as those who had “never smoked” [4].

Body mass index (BMI). Children were invited to write down their height and weight in country-appropriate units (cm vs. inches, pounds vs. kg). However, all the values were (re)coded in cm and kg, respectively. Evidence from Estonian adolescents supports the use of prevalence rates for overweight and obesity derived from self-reported measures as fairly accurate proxies, particularly when actual measurements are not available [24]. Thus, BMI was computed based on self-reported measures of height and weight for each adolescent. Adolescents were classified into four categories: “thin”, “normal weight”, “overweight”, and “obese”, according to the age- and sex-specific cut-offs recommended by the International Obesity Task Force (IOTF) [25]. We considered healthy weight or the ideal health behavior to be between “thin” and “normal weight” [4].

Physical activity. Physical activity was assessed by asking participants, “How often do you usually exercise in your free time to the point where you get out of breath or sweat?” Adolescents were given seven response options, ranging from “every day” to “never”. Previous studies have demonstrated strong reliability and validity in assessing physical activity [26,27]. Being physically active or the ideal health behavior was defined as adolescents reporting “exercising every day” [4].

Healthy Diet. A healthy diet was assessed based on the frequency of fruit, vegetable, sweets, and soft drink intake during the week. Adolescents were provided with seven response options, ranging from “never” to “more than once a day”. We defined a healthy diet or the ideal health behavior as adolescents reporting consuming fruits and vegetables “at least once a day or more” and sweets and soft drinks intake “at most once a week” [4].

Mental Health. A composite score of mental health from the HBSC Symptom Checklist (HBSC-SCL) was represented by mental health complaints (feeling low, feeling irritable, nervousness, and sleep difficulties) [20]. Responses range from 1 (‘about every day’) to 5 (‘rarely or never’) [21]. Higher scores reflect better mental health. Adolescents were categorized as healthy by “Rarely or never to about every week” and unhealthy by “about every day to more than once/week”. Haugland and Wold [28], in their quantitative analysis, demonstrated acceptable test–retest reliability for the HBSC symptom scale as a whole (Pearson r = 0.79) and slightly lower reliability for individual symptoms (Pearson r = 0.61 to 0.76).

Well-being. Well-being was composed of life satisfaction and self-reported health. Life satisfaction was assessed based on the ladder life scale. The ladder numbers correspond to 0–10 from bottom to top, with 10 at the top representing the best life and 0 at the bottom representing the worst life. Life satisfaction below 7 was considered low and above 7 was considered high. Self-reported health was assessed on a four-point scale (excellent, good, fair, or poor). We considered adolescents to be healthy when they answered excellent or good and unhealthy when they answered fair or poor. A previous study demonstrated the good reliability of the question regarding life satisfaction in adolescents [29]. In addition, analyses of past HBSC surveys have shown self-reported health to be associated with the general health item and the HBSC symptom checklist [30].

Socioeconomic status. Socioeconomic status was assessed by the Family Affluence Scale (FAS). Based on six questions, the scale had a range of 0 to 13 points. The highest socioeconomic position was indicated with a score of 13. By aggregating the responses, a continuous variable was generated to facilitate statistical analysis [31].

Statistical analysis. The data in this study are reported as counts and percentages for categorical variables and means and standard deviations for continuous variables. An independent-sample t-test was performed to compare the mean BMI and socio-economic status between genders. The Chi-square test or Mann–Whitney U test was performed to examine the associations between the frequency of cardiovascular health behaviors and gender, and mental health and gender. The multinominal regression was performed to examine the predictive probabilities of healthy mental health. Age, socioeconomic status, and gender were included in model 1 to examine predictive probabilities of characteristics of study participants to healthy mental health. In model 2, the predictive probabilities of health behaviors were examined separately for healthy mental health by including smoking status, BMI status, physical activity status, and eating status (ideal vs. non-ideal). The ICHBs were included in model 3 to examine the predictive probabilities of ICHBs for healthy mental health. The significance level was set at *p* ≤ 0.05 for all analyses. Statistical analyses were performed in PASW 25.0 Statistics (Chicago, IL, USA).

## 4. Results

A total of 227,441 respondents were assessed in the 2017/2018 HBSC Survey in Europe and Canada (112,333 boys and 115,108 girls). Among this total sample, 12,934 adolescents were from the Baltic countries, with 4725 (36.5%) from Estonia, 3797 (29.4%) from Lithuania, and 4412 (34.1%) from Latvia. The percentage of representation of Estonia, Lithuania, and Latvia involved in the 2018 HBSC study was 2.0%, 1.6%, and 1.8%, respectively. The response rates were 85.7%, 80.7%, and 73.5%, respectively. The sample included 4431 adolescents (34.3%) in the 11-year-old class group, 4386 adolescents (33.9%) in the 13-year-old class group, and 4066 adolescents (31.4%) in the 15-year-old class group. Out of the 12,934 adolescents, 6471 (50.0%) were male, and 6463 (50.0%) were female. There was no statistically significant difference in age among boys and girls (*p* = 0.075). However, the boy had a high BMI (*p* < 0.001) and the highest socioeconomic position compared to girls (*p* < 0.001, Table 1).

Overall, the majority of participants were non-smokers (71.2%) and had a normal BMI for their age and gender (71.1%). However, a small proportion of adolescents were found to have a healthy diet (21.5%), and only 4.5% of the total sample adhered to physical activity guidelines. The frequency of non-smokers, non-overweight individuals, and those with a healthy diet was lower among boys compared to girls, while boys were more physically active than girls (*p* < 0.05 for all, Table 1).

**Figure 1:**
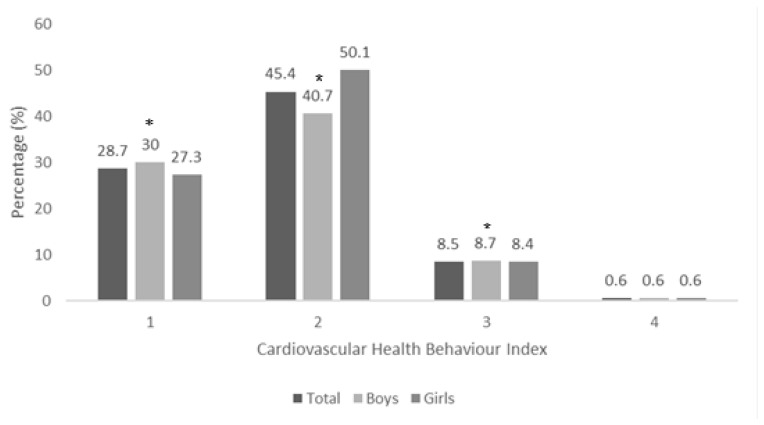
Differences in ICHBs between sexes. A chi-square test was used to analyze the data. * *p* < 0.05 indicates a significant difference between boys and girls.

Overall, 3706 (28.7%) had one ICHB, 5871 (45.4%) had two ICHBs, 1101 (8.5%) had three ICHBs, and 81 (0.6%) had four ICHBs. There was a significant statistical difference between cardiovascular health behavior status (unhealthy vs. health) and gender (*p* < 0.005). The frequency of one ICHB or three ICHBs was higher in boys than girls, while the frequency of two ICHBs was higher in girls compared to boys. Conversely, the frequency of four ICHBs remained unchanged (Figure 1).

Overall, less than half of the adolescents exhibited some negative mental health complaints. Also, mean life satisfaction was 7.7 ± 1.1. Girls had a higher frequency of negative mental health complaints than boys. There was a significant statistical difference between mental health parameters (unhealthy vs. health) and gender (*p* < 0.005). More details are described in Table 1.

Among boys, a higher frequency of mental health complaints was observed in those with non-ideal smoking behavior compared to those with ideal smoking behavior (*p* < 0.05). A higher frequency of irritability and feelings of nervousness were observed in those with non-ideal BMI compared to those with ideal BMI (*p* < 0.05). No statistically significant differences in mental health complaints were found based on physical activity status or healthy diet (*p* > 0.05). Among girls, a higher frequency of mental health complaints was also observed in those with non-ideal smoking behavior compared to those with ideal smoking behavior (*p* < 0.05), as well as in those with non-ideal BMI compared to those with ideal BMI (*p* < 0.05). No statistically significant differences in mental health complaints were found based on physical activity status (*p* > 0.05). However, a higher frequency of mental health complaints, except for irritability, was observed in those with a non-ideal healthy diet compared to those with an ideal healthy diet (*p* < 0.05). More details are described in Table 2.

Increased age was associated with lower odds of feeling low (OR = 0.83, 95% CI: 0.81–0.86), irritability (OR = 0.87, 95% CI: 0.85–0.89), and feeling nervous (OR = 0.82, 95% CI: 0.80–0.85). Conversely, age was associated with higher life satisfaction (OR = 1.22, 95% CI: 1.19–1.25). Boys had significantly higher odds than girls of feeling low (OR = 2.58, 95% CI: 2.35–2.89) and irritability (OR = 1.96, 95% CI: 1.81–2.12). However, boys had lower odds of life satisfaction (OR = 0.79, 95% CI: 0.73–0.85). Non-ideal smoking behavior was associated with lower odds of feeling low (OR = 0.52, 95% CI: 0.47–0.57), irritability (OR = 0.53, 95% CI: 0.49–0.58), and feeling nervous (OR = 0.50, 95% CI: 0.45–0.54). Non-ideal BMI showed mixed results, with higher odds for life satisfaction (OR = 1.25, 95% CI: 1.13–1.37) but lower odds for overall health (OR = 0.69, 95% CI: 0.62–0.77). Non-ideal physical activity was associated with lower health perception (OR = 0.65, 95% CI: 0.55–0.77) but higher life satisfaction (OR = 1.38, 95% CI: 1.23–1.56). Adolescents with one ICHB component had significantly lower odds of feeling low (OR = 0.28, 95% CI: 0.13–0.62), feeling nervous (OR = 0.26, 95% CI: 0.13–0.55), and sleep difficulties (OR = 0.29, 95% CI: 0.14–0.61). Those with two ICHB components also showed a protective effect against mental health issues but with slightly weaker odds. Interestingly, having three ICHB components was not significantly associated with most mental health outcomes, except for sleep difficulties (OR = 0.47, 95% CI: 0.22–0.98). Four ICHB components served as the reference group. More details are described in Table 3.

## 5. Discussion

The main results of our study revealed three key associations: (1) boys exhibited fewer ideal cardiovascular health behaviors (ICHBs) but reported fewer mental health issues compared to girls; (2) individuals who were less likely to engage in non-ICHB behaviors, such as smoking, high BMI, and low physical activity, tended to report better mental health; and (3) a higher likelihood of having a greater number of ICHBs was associated with better mental health compared to lower ICHB metrics. Hence, maintaining a healthy lifestyle appears to offer protection against mental health issues in adolescents from Baltic countries. These findings hold significant importance, considering the substantial burden of physical noncommunicable diseases linked to lifestyle behaviors. There is an urgent requirement to improve our comprehension of the complex links between the mental and physical health encounters of adolescents. To the best of our knowledge, this study represents the first attempt to examine the association between the accumulation of ICHBs and mental health or well-being in adolescents from Baltic countries.

In our study, the prevalence of non-smoking was 71.2%, ideal BMI was 71.1%, ideal physical activity was 13.4%, and ideal diet was 21.5%. In a study carried out in Portugal [7], the prevalence of non-smokers, physically active individuals, and those adhering to a healthy diet was greater compared to our study (93.0%, 33.0%, and 46.0%, respectively), while the prevalence of non-overweight individuals was similar (73%) to our findings. Another study conducted in Western Austria and South Tyrol [6] reported a similar prevalence of non-smokers (70.4%) compared to our findings, whereas the prevalence of non-overweight individuals was slightly higher (78.3%), with a greater proportion of physically active individuals (42.5%) and a lower prevalence of adherence to a healthy diet (8.1%) compared to our study. Additionally, boys exhibited fewer health behaviors overall, but they had the highest frequency of physical activity compared to girls. In contrast with our findings, Agostinis-Sobrinho [7,8] demonstrated in studies conducted in Portugal that boys were more physically active than girls. This confirms the differences in health behaviors among Baltic adolescents in comparison to the overall European context [32]. In addition, there are gender differences related to lifestyle habits with tendencies declining among boys [33,34].

Regarding mental health, the frequency of feeling low was 20.7%, irritability was 28.1%, nervousness was 24.5%, and difficulties in getting to sleep was 24.3%. In addition, mean life satisfaction was 7.7 ± 1 and 83.1% had good to excellent self-reported health. Findings from the 2017/2018 HBSC survey in Europe and Canada demonstrated frequencies slightly smaller compared to our findings for feeling low (18%) and irritability (25%), similar to nervousness (25%) and difficulties in getting to sleep (24%). Moreover, most adolescents felt satisfied with their lives, with an overall score of 7.8 out of 10 [11], which is similar to our findings. Lastly, the HBSC average ranged from 32–43% for self-reported health [35]. The highest frequency of self-reported health may be explained by the fact that we considered good and excellent answers to self-reported health while the HBSC considered only excellent answers to self-reported health. In addition, we observed that mental health was more favorable in boys compared to girls. In the same way, the HBSC reported that girls experienced individual health complaints more often than boys [11]. This aligns with the results of a recent study [36] where girls showed significantly higher scores than boys across a range of mental health problems and subjective well-being measures. Therefore, our findings underscore the disparity between low mental health indicators in the Baltic countries compared to European data, as well as the disparity between genders. This highlights the greater need for public policies addressing mental health in adolescents in Baltic countries.

According to the current literature, compliance with ICHBs has been shown to be an important measure for maintaining cardiovascular health, preventing diseases, and promoting overall well-being at both individual and population levels [7,37,38,39,40]. Our results show that as the number of ICHBs increases, the better the results of mental health complaints, suggesting that enhancing cardiovascular health behavior during adolescence is likely to have positive effects on mental health. Corroborating our findings, Maenhout et al. [41] demonstrated in 1037 adolescents that all healthy lifestyle behaviors were associated with at least one mental health outcome. Additionally, our findings align with larger studies that have highlighted the adverse effects of unhealthy behaviors on mental health in adolescents [41,42]. Similarly, a recent longitudinal study suggested that low levels of physical activity, an unhealthy diet pattern, and insomnia in adolescence were associated with psychological distress in young adulthood [43].

A previous study demonstrated that the accumulation of ICHBs (four behaviors) is linked to cardiorespiratory fitness [7] and mental health in adolescents [44,45]. Additionally, previous studies have demonstrated that better ICHB is already associated with a lower inflammatory profile in adolescence [46]. Moreover, it is recognized that multiple factors influence mental health during adolescence, with social determinants playing a particularly significant role [47,48]. Therefore, recognizing this relationship, studies assessing the interrelationship among ICHBs, inflammation, and social determinants are necessary.

A major strength of the study is that it is based on large-scale data from a nationally representative sample of Baltic countries. Also, this study determined health behavior from the ideal health behaviors index proposed by AHA. This metric is utilized globally and across various populations to assess and monitor the prevalence and incidence of cardiovascular health status. This enables the extrapolation of results to other populations. On the other hand, this study has some limitations. First, all data were self-reported, so measurement bias should be considered. Second, data collection was cross-sectional and did not allow for causal inferences. For this reason, longitudinal cohort studies are necessary to provide better information over time on the associations between ICHBs and mental health.

## 6. Conclusions

In conclusion, the boys showed lower metrics of cardiovascular health behaviors and negative mental health compared to girls. Additionally, our study showed that the accumulation of cardiovascular health behavior metrics was associated with negative mental health and well-being compared to ideal cardiovascular health behaviors. These findings suggest important public health policy implications for Baltic countries, including the promotion of healthy eating habits, increased physical activity, and the implementation of school-based programs that encourage healthy lifestyles. Public health policies should also advocate for the balanced use of digital technology and promote activities that limit screen time. By focusing on these areas, policymakers can create environments that support the overall well-being of adolescents. Consequently, these public health policies will enhance the mental well-being of adolescents and ensure their healthy development into adulthood in Baltic countries.

## Figures and Tables

**Table 1:** Characteristics of study participants.

	Total	Boys (n = 6471)	Girls (n = 6463)
Age (years), mean ± SD	13.6 ± 1.6	13.7 ± 1.6	13.6 ± 1.6
Body mass index (kg/m^2^), mean ± SD	19.8 ± 3.6	20.0 ± 3.7	19.6 ± 3.4 *
Socioeconomic Status, mean ± SD median (CI 95%)	7.5 ± 2.5 8.0 (6.0–9.0)	7.6 ± 2.4 8.0 (6.0–9.0)	7.3 ± 2.5 * 7.0 (6.0–9.0)
Cardiovascular health behaviors			
Non-smokers, n (%)	9214 (71.2)	4454 (68.8)	4760 (73.7) ^#^
Non-overweight, n (%)	9199 (71.1)	4272 (66.0)	4927 (76.2) ^#^
Physically active, n (%)	1737 (13.4)	1057 (16.3)	680 (10.5) ^#^
Healthy diet, n (%)	584 (4.5)	244 (3.8)	340 (5.3) ^#^
Mental health			
Feel Low, n (%)	2671 (20.7)	863 (13.3)	1808 (28.0) ^#^
Irritable, n (%)	3632 (28.1)	1385 (21.4)	2247 (34.8) ^#^
Nervous, n (%)	3170 (24.5)	1160 (17.9)	2010 (31.1) ^#^
Sleep difficulty, n (%)	3141 (24.3)	1287 (19.9)	1854 (28.7) ^#^
Low life satisfaction, n (%)	4793 (38.4)	2288 (35.4)	2685 (41.5) ^#^
Low self-reported health, n (%)	2189 (16.9)	855 (13.2)	1334 (20.6) ^#^

* The independent-samples *t*-test; ^#^ The chi-square test.

**Table 2:** The frequency of ICHBs and mental health of study participants stratified by gender.

	Boys								Girls							
	Feel Low		Irritability		Feel Nervous		Difficulty in Getting to Sleep		Feel Low		Irritability		Feel Nervous		Difficulty in Getting to Sleep	
	n (%)	*p*	n (%)	*p*	n (%)	*p*	n (%)	*p*	n (%)	*p*	n (%)	*p*	n (%)	*p*	n (%)	*p*
Smoking behaviors																
Ideal	522 (11.9)	*p* < 0.001	847 (19.2)	*p* < 0.001	682 (15.5)	*p* < 0.001	788 (17.9)	*p* < 0.001	1093 (23.1)	*p* < 0.001	1398 (29.6)	*p* < 0.001	1224 (25.9)	*p* < 0.001	1171 (24.8)	*p* < 0.001
Non-ideal	321 (17.3)		505 (27.1)		443 (23.9)		466 (25.1)		683 (42.7)		812 (50.7)		761 (47.6)		645 (40.3)	
BMI																
Ideal	535 (12.7)	*p* < 0.05	864 (20.4)	*p* < 0.001	709 (16.8)	*p* < 0.001	824 (19.4)	0.066	1327 (27.1)	*p* < 0.001	1669 (34.1)	*p* < 0.001	1514 (30.9)	*p* < 0.05	1387 (28.3)	0.031
Non-ideal	208 (14.7)		350 (24.7)		291 (20.6)		308 (21.8)		327 (32.4)		409 (40.7)		353 (34.9)		320 (31.7)	
Physical activity																
Ideal	139 (13.4)	0.989	208 (20.0)	0.187	186 (17.9)	0.933	205 (19.7)	0.728	192 (28.6)	0.836	229 (34.0)	0.589	198 (29.4)	0.290	214 (31.8)	0.081
Non-ideal	710 (13.5)		1159 (21.9)		955 (18.1)		1068 (20.2)		1601 (28.1)		1998 (35.1)		1794 (31.5)		1621 (28.5)	
Healthy diet																
Ideal	29 (12.2)	0.605	43 (18.0)	0.195	38 (15.8)	0.390	40 (16.7)	0.211	78 (23.1)	0.039	112 (33.1)	0.502	89 (26.3)	*p* < 0.05	81 (24.0)	*p* < 0.05
Non-ideal	830 (13.6)		1332 (21.7)		1116 (18.2)		1236 (20.2)		1724 (28.4)		2127 (35.1)		1913 (31.5)		1767 (29.1)	

The chi-square test was performed to assess the association between ideal and non-ideal frequency.

**Table 3:** Multinominal regression to examine the association between the accumulation of ICHBs and mental health and well-being in adolescents from Baltic countries.

			Feel Low	Irritability	Feel Nervous	Sleep Difficulty	Life Satisfaction	Self-Reported Health
Model 1	Health	Age, OR (CI 95%)	0.83 (0.81–0.86; *p* < 0.001)	0.87 (0.85–0.89; *p* < 0.001)	0.82 (0.80–0.85; *p* < 0.001)	0.94 (0.92–0.96; *p* < 0.001)	1.22 (1.19–1.25; *p* < 0.001)	0.87 (0.81–0.89; *p* < 0.001)
		Socioeconomic status, OR (CI 95%)	*p* > 0.05	1.03 (1.02–1.05; *p* < 0.001)	*p* > 0.05	1.02 (1.00–1.03; *p* < 0.05)	0.88 (0.86–0.89; *p* < 0.001)	1.10 (1.08–1.12; *p* < 0.001)
		Gender						
		Boys, OR (CI 95%)	2.58 (2.35–2.89; *p* < 0.001)	1.96 (1.81–2.12; *p* < 0.001)	2.11 (1.94–2.29; *p* < 0.001)	1.61 (1.48–1.75; *p* < 0.001)	0.79 (0.73–0.85; *p* < 0.001)	1.70 (1.55–1.87; *p* < 0.001)
		Girls	Reference	Reference	Reference	Reference	Reference	Reference
Model 2	Health	Smoking behavior						
		Non-ideal, OR (CI 95%)	0.52 (0.47–0.57; *p* < 0.001)	0.53 (0.49–0.58; *p* < 0.001)	0.50 (0.45–0.54; *p* < 0.001)	0.58 (0.53–0.64; *p* < 0.001)	2.10 (1.93–2.29; *p* < 0.001)	0.56 (0.50–0.62; *p* < 0.001)
		Ideal	Reference	Reference	Reference	Reference	Reference	Reference
		BMI						
		Non-ideal, OR (CI 95%)	0.90 (0.8–1.00; *p* < 0.05)	0.84 (0.76–0.93; *p* < 0.001)	0.88 (0.80–0.98; *p* < 0.05)	0.90 (0.81–1.00; *p* < 0.05)	1.25 (1.13–1.37; *p* < 0.001)	0.69 (0.62–0.77; *p* < 0.001)
		Ideal	Reference	Reference	Reference	Reference	Reference	Reference
		Physical activity						
		Non-ideal, OR (CI 95%)	*p* > 0.05	0.83 (0.73–0.94; *p* < 0.001)	0.84 (0.74–0.96; *p* < 0.001)	*p* > 0.05	1.38 (1.23–1.56; *p* < 0.001)	0.65 (0.55–0.77; *p* < 0.001)
		Ideal	Reference	Reference	Reference	Reference	Reference	Reference
		Healthy diet						
		Non-ideal, OR (CI 95%)	*p* > 0.05	*p* > 0.05	*p* > 0.05	*p* > 0.05	1.87 (1.51–2.31; *p* = 0.00)	0.62 (0.46–0.83; *p* = 00)
		Ideal	Reference	Reference	Reference	Reference	Reference	Reference
Model 3	Health	ICHB						
		1 component, OR (CI 95%)	0.28 (0.13–0.62; *p* < 0.001)	0.35 (0.19–0.66; *p* < 0.001)	0.26 (0.13–0.55; *p* < 0.001)	0.29 (0.14–0.61; *p* < 0.001)	5.24 (2.83–9.70; *p* < 0.001)	0.09 (0.02–0.38; *p* < 0.001)
		2 components, OR (CI 95%)	0.45 (0.20–0.97; *p* < 0.05)	0.52 (0.28–0.96; *p* < 0.05)	0.40 (0.19–0.83; *p* < 0.05)	0.38 (0.18–0.80; *p* < 0.05)	3.03 (1.63–5.60; *p* < 0.001)	0.15 (0.04–0.60; *p* < 0.001)
		3 components, OR (CI 95%)	*p* > 0.05	*p* > 0.05	*p* > 0.05	0.47 (0.22–0.98; *p* < 0.05)	1.91 (1.02–3.57; *p* < 0.05)	0.24 (0.06–1.00; *p* < 0.05)
		4 components, OR (CI 95%)	Reference	Reference	Reference	Reference	Reference	Reference

Reference category: unhealthy; SES, socioeconomic status; OR, odds ratio; CI, Confidence interval.

## Data Availability

The data presented in this study are available on request from the corresponding author due to a secondary analysis of anonymized data.

## Abbreviations

The following abbreviations are used in this manuscript:

AHA	American Heart Association
BMI	Body mass index
CI	Confidence interval
EU	Europe
FAS	Family Affluence Scale
HBSC	Health Behaviors in School-aged Children
HBSC-SCL	HBSC Symptom Checklist
ICHB	Cardiovascular health behavior
OR	Odds ratio
SES	Socioeconomic status
WHO	World Health Organization
